# Dynamics of natural killer cell function upon recurrent stimulation

**DOI:** 10.1002/btpr.70100

**Published:** 2026-01-13

**Authors:** Jennifer One, Janani Narayan, Frank Cichocki, Wei‐Shou Hu, Samira M. Azarin

**Affiliations:** ^1^ Department of Biomedical Engineering University of Minnesota Minneapolis Minnesota USA; ^2^ Department of Chemical Engineering and Materials Science University of Minnesota Minneapolis Minnesota USA; ^3^ Department of Medicine University of Minnesota Minneapolis Minnesota USA

**Keywords:** biomanufacturing, cytotoxicity, growth, metabolism, natural killer cells

## Abstract

Natural killer (NK) cells have shown potential for allogeneic cell‐based cancer immunotherapies. For development of economical off‐the‐shelf allogeneic therapies, maximal expansion of the NK cells from each donor must be achieved while maintaining efficacy and uniformity of the cell product. The standard method for robust expansion utilizes weekly stimulation with engineered feeder cells derived from the K562 cell line. However, the effects of repeated stimulation on NK cell growth, metabolism, and function are not well understood. In this study, we demonstrated a distinct shift in growth kinetics and metabolism around week 3–4 of repeated K562 feeder cell stimulation, followed by a change in cytokine secretion and killing ability. Seahorse metabolic flux assays and transcriptomics suggested a transition from glycolytic metabolism to oxidative metabolism after the first week of stimulation, but the shift in growth kinetics generally correlated to reduced metabolic activity. Collectively, these results indicate that serial stimulation sustains large‐fold NK cell expansion that can be exploited for NK cell therapy; however, this expansion has important impacts on NK cell growth, metabolism, and function. Careful characterization is critical when developing large‐scale biomanufacturing processes to ensure efficacy of the final cellular product.

## Graphical abstract

Weekly stimulation of peripheral blood‐derived NK cells with engineered K562 feeder cells resulted in two distinct growth phases, shifting around Day 21–26. Cells in growth phase 2 exhibited slower growth, reduced metabolic activity, and lower cytotoxicity than cells from growth phase 1, which has implications for NK cell biomanufacturing.

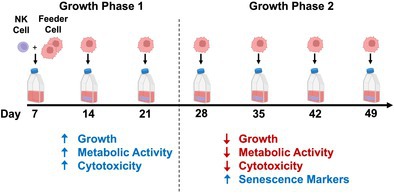

## 1 INTRODUCTION

Recent clinical studies have demonstrated the promise of allogeneic adoptive natural killer (NK) cell transfer, in which the cells from one donor can be administered to many patients, as an emerging cell therapy for cancer.^1^, ^2^, ^3^, ^4^ NK cells are part of the innate immune system, which initiates the first line of defense against infection and foreign pathogens, and they play a crucial role in immunosurveillance of malignancies due to their cytotoxic and cytokine‐producing effector functions.^2^, ^5^ NK cells rely on a balance of activating and inhibitory receptors for activation and thus have the capacity to kill tumor cells without the requirement of advance priming, unlike T cells.^2^, ^5^ Furthermore, this ability allows NK cells to recognize tumor cells in the absence of major histocompatibility (MHC) class I molecules and antigen, which are often downregulated on cancer cells to prevent detection by T cells.^6^, ^7^, ^8^ Despite MHC class I molecule mismatch between donor and recipient cells, various studies have shown no induction of graft‐versus‐host disease upon adoptive transfer of NK cells,^2^, ^9^ making NK cells suitable for allogeneic cell therapy.

One challenge in the cost‐ and time‐efficient production of an allogeneic NK cell therapy is the ability to expand the primary cell source to obtain a sufficient number of cells. Reducing production costs through economies of scale while still ensuring product uniformity is essential. Clinical trials involving NK cells often call for high doses, up to 10^9^ cells/kg, for increased efficacy.^4^, ^10^, ^11^ Accordingly, to treat 100 patients from a single donor, the quantity needed is on the order of 10^13^ cells per manufacturing batch. Given that the number of cells taken from a donor is not enough to meet this demand, NK cells isolated from a single donor must be expanded through an efficient large‐scale biomanufacturing process to generate sufficient doses for an economical “off‐the‐shelf” therapy. Various methods have been utilized to activate and expand NK cells, including cytokine stimulation,^12^ monoclonal antibodies,^13^ bead‐tethered cytokines and receptors,^14^ and various feeder cell lines.^15^, ^16^, ^17^, ^18^, ^19^, ^20^, ^21^ The most significant expansion to date has been achieved with the use of engineered feeder cells, derived from the K562 leukemia cell line, which have been modified to express various co‐stimulatory ligands on the cell surface. Denman et al. found that K562 feeder cells expressing membrane‐bound IL‐21 and 41BBL enabled 10^4^‐fold NK cell expansion in 3 weeks, outperforming the expansion achieved with K562 feeder cells expressing membrane‐bound IL‐15, and observed no evidence of senescence for up to 6 weeks,^17^ thus making the use of K562 cells expressing membrane‐bound IL‐21 and 41BBL a promising feeder cell line to meet expansion requirements.

While feeder cell‐induced expansion of NK cells holds the potential to meet biomanufacturing standards for an economical off‐the‐shelf product, there is a need for dynamic characterization to comprehensively assess the function and metabolism of the expanded cells to better understand the tradeoffs between cell expansion and quality. Chronic antigen stimulation has been found to induce genome‐wide epigenetic programming and dysfunction in T cells, leading to the concern that this may also occur in NK cells.^22^, ^23^ Therefore, from a bioprocessing perspective, determining shifts in functionality and metabolism over time could be key in establishing whether the repeated stimulation necessary to achieve large cell yields compromises product quality in terms of NK cell function.

In this study, the expansion potential of peripheral blood NK cells upon repeated stimulation with K562 feeder cells was evaluated by measurement of growth kinetics and cell cycle status. NK cell function was investigated by assessing the levels of perforin, granzyme, and IFN‐γ, which aid in the killing process, as well as tumor killing kinetics against a broad array of solid tumor cell lines. Furthermore, our data was supplemented by Seahorse metabolic flux assays to assess mitochondrial metabolism and glycolytic metabolism dynamics over time. RNA‐sequencing was used to identify transcriptional changes occurring during the extended culture and to explore signaling pathways active during the different growth phases of the culture. These studies revealed key alterations in NK cell growth and function upon repeated stimulation, highlighting the need for further characterization of biomanufacturing processes for large‐scale production of NK cells to ensure product uniformity and clinical efficacy.

## 2 MATERIALS AND METHODS

### 2.1 Cell culture and NK cell expansion

Naïve K562 cells were obtained from ATCC, and K562 feeder cells engineered to express CD137L (41BBL) and membrane‐bound IL‐21^17^ were kindly provided by Dr. Dean Lee (MD Anderson Cancer Center, University of Texas). K562 medium formulation included RPMI 1640 Medium with L‐glutamine (Corning), 1% penicillin/streptomycin (PS, Gibco), and 10% heat‐inactivated fetal bovine serum (FBS, Hyclone). K562 feeder cells were irradiated at 10,000 rads and cryopreserved in 90% FBS and 10% DMSO (Sigma) prior to thawing and co‐culture with NK cells as feeder cells. Peripheral blood mononuclear cells were obtained from Memorial Blood Center (Minneapolis, MN) using Ficoll‐Paque Premium (GE Healthcare). NK cell isolation was performed using negative selection with the EasySep Human NK Cell Enrichment Kit (STEMCELL Technologies). NK cells were cultured overnight in B0 medium^24^, ^25^ supplemented with 100 U/mL IL‐2 (Proleukin) to recover from magnetic bead sorting. B0 medium formulation included a 2:1 (vol: vol) mix of Dulbecco's Modification of Eagle's Medium (DMEM) with 4.5 g/L glucose, L‐glutamine, and sodium pyruvate and Ham's F12 Medium (Corning). Supplements included 20 μM 2‐mercaptoethanol (Gibco), 50 μM ethanolamine (Sigma), 10 μg/mL ascorbic acid (Sigma), 1.6 ng/mL sodium selenite (Sigma), 1% penicillin/streptomycin, and 20% heat‐inactivated human AB serum (Valley Biomedical). NK cells were collected the following day for functional and metabolic measurements prior to stimulation with a 1:2 ratio of NK cells to irradiated K562 feeder cells in B0 medium supplemented with 50 U/mL IL‐2. A schematic for the expansion process is shown in Figure S1A. Seven days after initial stimulation, and weekly thereafter, serial stimulation was conducted by re‐seeding NK cells at 1.25 × 10^5^ cell/mL in fresh medium and re‐stimulating with K562 feeder cells at a 1:1 ratio. Spent medium samples were removed for analysis immediately prior to each round of re‐stimulation. In the first week of culture, half‐medium changes were performed at day 3 and day 5, and during subsequent weeks cells were re‐seeded to 2.5e5 cells/mL every 2–3 days between rounds of stimulation (Figure S1A,B).

### 2.2 Growth kinetics and cell cycle analysis

Cell counting was performed every 2–3 days using the Countess II Automated Cell Counter after adding a 1:1 ratio of Trypan Blue (Gibco) to the sample. To determine the cutoff between growth phases, cumulative total cell number data were log_2_‐transformed to visualize the breakpoint between growth phases by a clear shift in slope. The doubling time for each growth phase was determined from the inverse of the slope of the log_2_‐transformed data.

For cell cycle analysis, NK cells were collected weekly prior to re‐stimulation. Cells were washed and stored in 100% Ethanol (Fisher Scientific) at −20°C. Once samples at all timepoints were collected, cells were rehydrated in PBS for 15 min at room temperature and washed in FACS buffer (PBS supplemented with 2% FBS and 2 mM EDTA). Cells were stained with a 1:20 dilution of 1 mg/mL propidium iodide (Life Technologies) in FACS buffer for 15 min at room temperature in the dark. Samples were immediately analyzed on the LSR II H4710 instrument (BD Biosciences). ModFit LT 4.1.7 was utilized to analyze cell cycle distribution in the G0/G1, G2/M, and S Phases.

### 2.3 Seahorse metabolism and oxygen uptake rate

Glycolysis and oxidative mitochondrial metabolism were concurrently analyzed using the Seahorse XF Cell Mito Stress Test Kit (Agilent Technologies) with modifications as described in a previous study.^24^ NK cells were plated at 1 × 10^6^ per well in at least triplicate for analysis using the XF^e^ 24 Extracellular Flux Analyzer (Agilent Technologies). Glucose, oligomycin, FCCP, rotenone, and antimycin A were sequentially injected to measure oxygen consumption rate (OCR) and extracellular acidification rate (ECAR). Basal respiration, maximal respiration, and ATP‐linked respiration were calculated from the OCR values according to the manufacturer's instructions. Similarly, glycolysis, glycolytic reserve, and glycolytic capacity were measured from the ECAR values according to the manufacturer's instructions. Oxygen uptake rate (OUR) was calculated from the average basal OCR values for each donor at each timepoint.

### 2.4 Glucose and glutamine consumption and lactate production

Prior to each medium change, the spent medium was removed and stored at −80°C. For analysis, medium samples were thawed and then centrifuged to remove any cell debris. 100 μl of cell‐free supernatant was added to a 96‐well plate (Corning) for quantification of glucose, glutamine, and lactate concentrations through enzymatic analysis using the YSI 2950D‐3 Biochemistry Analyzer (Xylem Inc.) according to the manufacturer's instructions. Cumulative moles of glucose and glutamine consumed and moles of lactate produced were determined by adjusting for dilution according to the ratio of the volumes of new and old medium at each medium change, then calculating the change in concentration between timepoints. The ratio of lactate production to glucose consumption and the ratio of glutamine consumption to glucose consumption were calculated for each donor and plotted for various timepoints.

### 2.5 RNA sequencing

Total RNA was isolated from NK cells at day 0, 7, and 35 using the Qiagen™ RNeasy Mini Kit. The Illumina TruSeq® Stranded mRNA Sample Prep Kit protocol was used to purify poly‐A containing mRNA molecules from the total RNA and create sequence libraries. Sequence libraries of each sample were pooled and sequenced using the Illumina HiSeq 2500 instrument (High Output, 125 bp, Paired Reads, v4) at the University of Minnesota Genomics Center. Low quality ends and adapter sequences were trimmed from the Illumina reads using Trimmomatic. Processed reads were mapped and aligned to the human reference genome (GRCh38.91) using Spliced Transcripts Alignment to a Reference (STAR) with default ENCODE parameter settings, except for ‘—runThreadN 16 —sjdbOverhang 76’. Reads Per Kilobase of transcript per Million mapped reads (RPKM) values were computed using Cufflinks and then normalized to transcripts per million (TPM). Using R Studio, TPM was then log_2_‐transformed before performing hierarchical clustering using the Euclidean distance and centroid linkage (UPGMC) method. Principal component analysis (PCA) was performed using the ‘ggplot2’ package in statistical software R (v 3.3.3).

Differential gene expression analysis was performed in R. Python software ‘htseq‐counts’ was utilized to extract the necessary raw read counts to upload into the ‘EdgeR’ package to determine differential gene expression. Gene expression was normalized with trimmed mean of M‐values (TMM) to adjust for falsely positive downregulated genes after lowly expressed genes (less than 6–7 counts) were filtered out. Negative binomial generalized linear models were then fitted prior to either implementing the quasi‐likelihood (QL) F‐test or likelihood ratio test for verifying differential gene expression. Transcripts which met the threshold *p*‐value with additional criteria of FDR ≤ 0.05 and fold change ≥ 2 were identified as differentially expressed in various pair‐wise comparisons. Gene Ontology (GO) analysis of the differentially expressed genes was performed using DAVID v6.8. Further visualizations of the data including the PCA plot and quadrant analysis for comparative analysis of pair‐wise comparisons were completed using the TIBCO Spotfire v7.6.1 OmicsOffice package.

### 2.6 Flow cytometry characterization

Clone numbers and additional details for antibodies used for analysis of marker expression in NK cells are listed in Table S1. For analysis of surface markers (CD56 and CD3), NK cells were stained with a 1:1000 dilution of Fixable Viability Dye eFluor780 (Thermo Fisher Scientific) for 10 min at 4°C in the dark. Cells were washed with FACS buffer (PBS supplemented with 2% FBS and 2 mM EDTA) prior to staining for 20 min at 4°C in the dark. Cells were then washed with FACS buffer prior to fixation in 2% paraformaldehyde (PFA). For intracellular staining of perforin and granzyme, NK cells were stained with identifying surface markers CD56 and CD3 using the surface staining protocol as described above. Upon fixation in 2% PFA, cells were incubated for 10 min at 37°C. NK cells were washed in FACS buffer and incubated in 1X permeabilization buffer, 10X permeabilization buffer (eBioscience) diluted in distilled water according to the manufacturer's instructions, for 10 min at 4°C. Cells were again washed with FACS buffer before incubating cells with intracellular antibodies for 30 min at 4°C. Samples were then washed and stored in FACS buffer prior to analysis. Flow cytometry data was acquired using the LSR II H4710 instrument (BD Biosciences) and analyzed with FlowJo v10 software.

### 2.7 NK cell effector function assay

To evaluate NK cell degranulation, NK cell samples were cultured alone or with naïve K562 target cells at a 1:2 ratio in 200 μL of B0 medium in a 96‐well round bottom plate. Cells were incubated with CD107a antibody (H4A3: BD Biosciences) for 1 h at 37°C prior to the addition of 20 μL of 1:100 GolgiPlug and 1:150 GolgiStop (BD Biosciences). Cells were subsequently incubated for 3.5 h at 37°C prior to being stained with Fixable Viability Dye eFluor780 (Thermo Fisher Scientific). Cells were stained for CD56 and CD3 and then fixed with 2% PFA as described above. To stain for interferon gamma (IFNγ), the steps for intracellular staining were followed as described earlier. Marker expression was analyzed with the LSR II H4710 instrument (BD Biosciences) and FlowJo v10 software. The magnitude of degranulation, as well as IFNγ production, were evaluated as the difference in percentage expression in NK cells co‐cultured with K562 cells relative to NK cells cultured alone.

### 2.8 Killing kinetics assay

A549, PANC‐1, and SKOV‐3 cells expressing NucLight Red (NLR) were utilized to assess the killing kinetics of NK cells from various donors over time. Each NLR target cell line was plated at a concentration of 5 × 10^3^ cells per well in a 96‐well clear bottom, black plate (Corning). Cells were allowed to attach overnight in DMEM supplemented with 10% FBS, 1X penicillin/streptomycin, and glutamine. The IncuCyte Zoom instrument (Essen BioScience) was used to quantify the number of target NLR cells per well. After counting the target cells, NK cells were added at a 10:1 effector to target ratio in B0 medium containing 50 U/mL IL‐2. Each condition was prepared in triplicate for each experiment. The number of viable target cells was tracked by imaging every 30 min over a period of 50 h using the IncuCyte Live Cell Analysis System. Quantification of live target cell number at each timepoint was performed using the IncuCyte software and normalized to the number of live cells at the initial timepoint as well as the live cells remaining in the control group containing only target cells.

### 2.9 Statistical analysis

Data were collected from three or four independent donors for each assay and expressed as the mean ± SD. Differences between groups within each growth phase or timepoint were determined by paired one‐tailed Student's *t* test, ANOVA analysis with Tukey's multiple comparisons tests, or a mixed effects model with Tukey's multiple comparisons tests as specified in the figure legends. The *p*‐values < 0.05 were considered statistically relevant.

## 3 RESULTS

### 3.1 Shift in growth kinetics observed during sustained NK cell expansion

**FIGURE 1:**
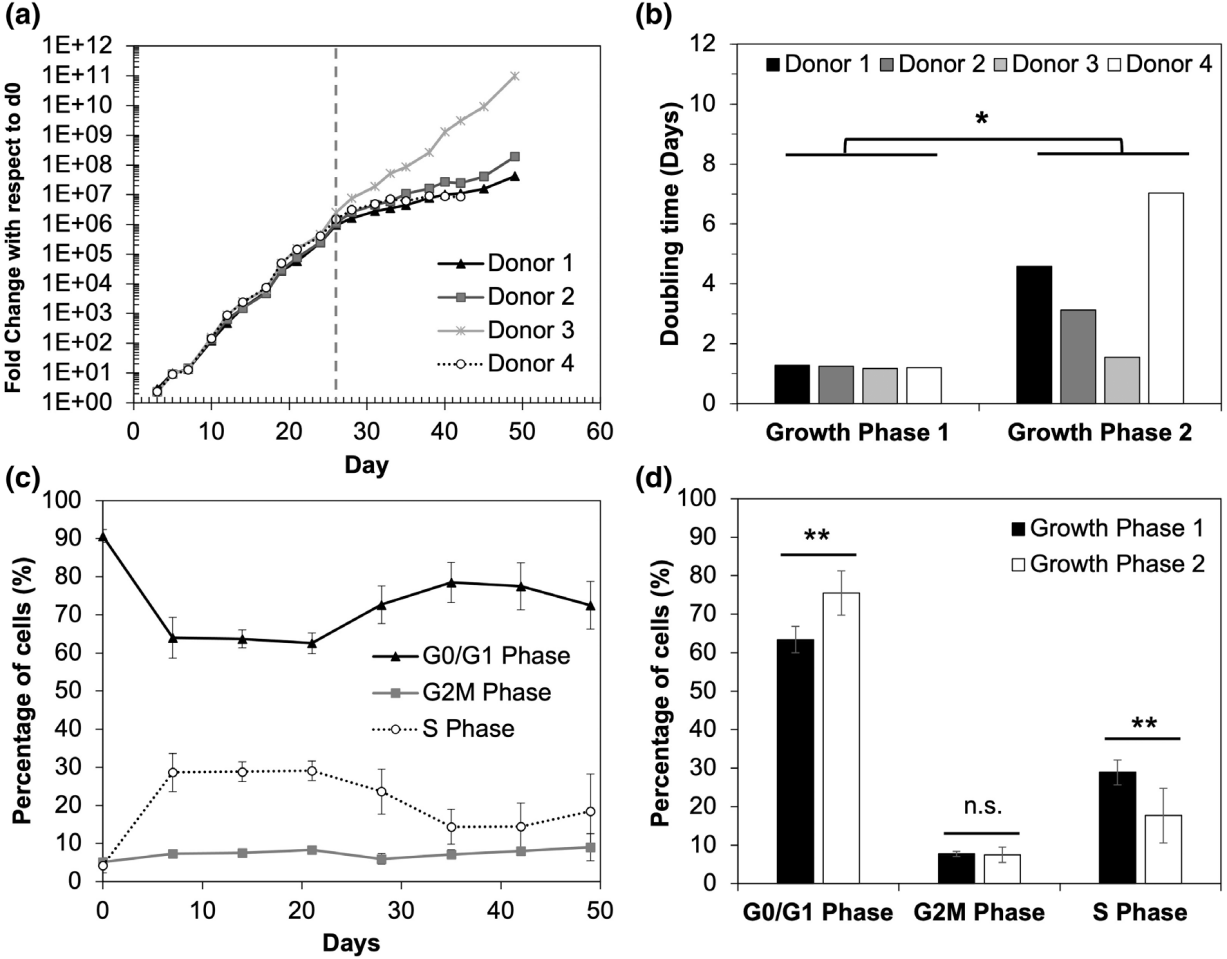
Characterization of growth kinetics during NK cell expansion. (a) Fold change in cell expansion over time for each donor (*n* = 4); dashed line indicates the transition to a slower growth phase at day 26. (b) Doubling time in days for growth phase 1 (prior to day 26) and growth phase 2 (after day 26) for each donor. * *p* < 0.05 for average of growth phase 2 doubling time values compared to growth phase 1 (Student's t test). (c) Average percentage of cells in each cell cycle phase (G0/G1, G2/M, and S phase) from flow cytometric analysis of PI staining across all donors at each timepoint. (d) Average percentage of cells in each cell cycle phase from (c) for the different growth phases (day 0 not included in growth phase 1). *p < 0.05, **p < 0.0001, n.s., not significant as determined via Student's t test.

NK cells from four donors were expanded via eight rounds of weekly stimulation with K562 feeder cells engineered to express membrane‐bound IL‐21 (Figure S1A,B). Cells from all four donors grew exponentially and all exhibited a shift to slower cell growth around day 26, although the growth slowdown was less pronounced in donor 3 (Figure 1a). The average doubling time for the four donors during the growth phase 1 was 1.23 ± 0.04 days (Figure 1b), which is the expected doubling time for healthy expanding NK cells in culture.^26^ During the growth phase 2, the average doubling time increased to 4.0 ± 2.3 days. Evaluation of cell cycle distribution via flow cytometric analysis of propidium iodide staining showed a statistically significant decrease in the percentage of cells in S phase and an increase in the percentage of cells in G0/G1 phase of the cell cycle after day 26 (Figure 1c,d). Purity, as measured by CD56 expression, remained high throughout the culture (Figure S1C). Average NK cell viability remained at approximately 80%–90% throughout culture, though donors 1 and 4 showed decreased viability in growth phase 2 before recovering at the end of the culture (Figure S1D). Donor 4 expansion slowed by day 42 and cells were only 48% viable, so expansion was not continued past day 42 for this donor. For the other three donors, average viability was 88 ± 3% at day 49 and growth had not yet plateaued, suggesting cells from these donors may have been able to continue expanding past the study endpoint of 49 days.

### 3.2 Changes in metabolism during NK cell expansion

**FIGURE 2:**
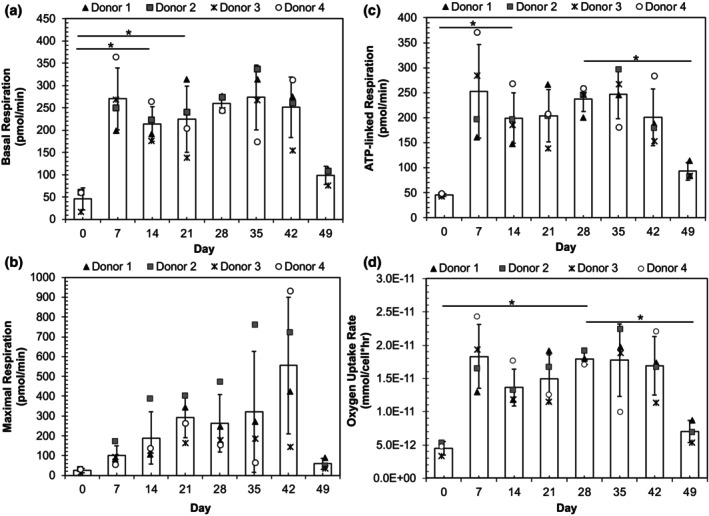
Characterization of mitochondrial metabolism during NK cell expansion. (a–c) Seahorse OCR profiles for each donor and average across all donors (white bars) for basal respiration (a), maximal respiration (b), and ATP‐linked respiration (c). (d) Specific oxygen consumption rate over time for each donor as well as the average across all donors. *p < 0.05 via a mixed effects model with Tukey's multiple comparisons tests.

NK cell metabolism was assessed by monitoring oxidative mitochondrial metabolism and glycolysis using Seahorse assays. The oxygen consumption rate (OCR) was measured at the basal state after the addition of oligomycin, which inhibits ATP synthesis and suppresses oxygen consumption, the maximal state after the addition of FCCP, which uncouples ATP synthesis from the electron transport chain, and the minimal state after addition of rotenone and antimycin A, which inhibit electron transfer in complexes I and III of the electron transport chain.^27^ The basal, maximal, and ATP‐linked respiration were all low prior to stimulation (day 0), increased after feeder cell activation, then dropped at the final timepoint (day 49) (Figure 2a–c). The specific oxygen consumption rate calculated from the OCR also showed an increase after feeder cell stimulation and a decrease on day 49 (Figure 2d). The roughly 4‐fold increase in specific oxygen consumption rate in the first week could be attributed to a switch from a relatively quiescent state to a rapidly proliferating state.

**FIGURE 3:**
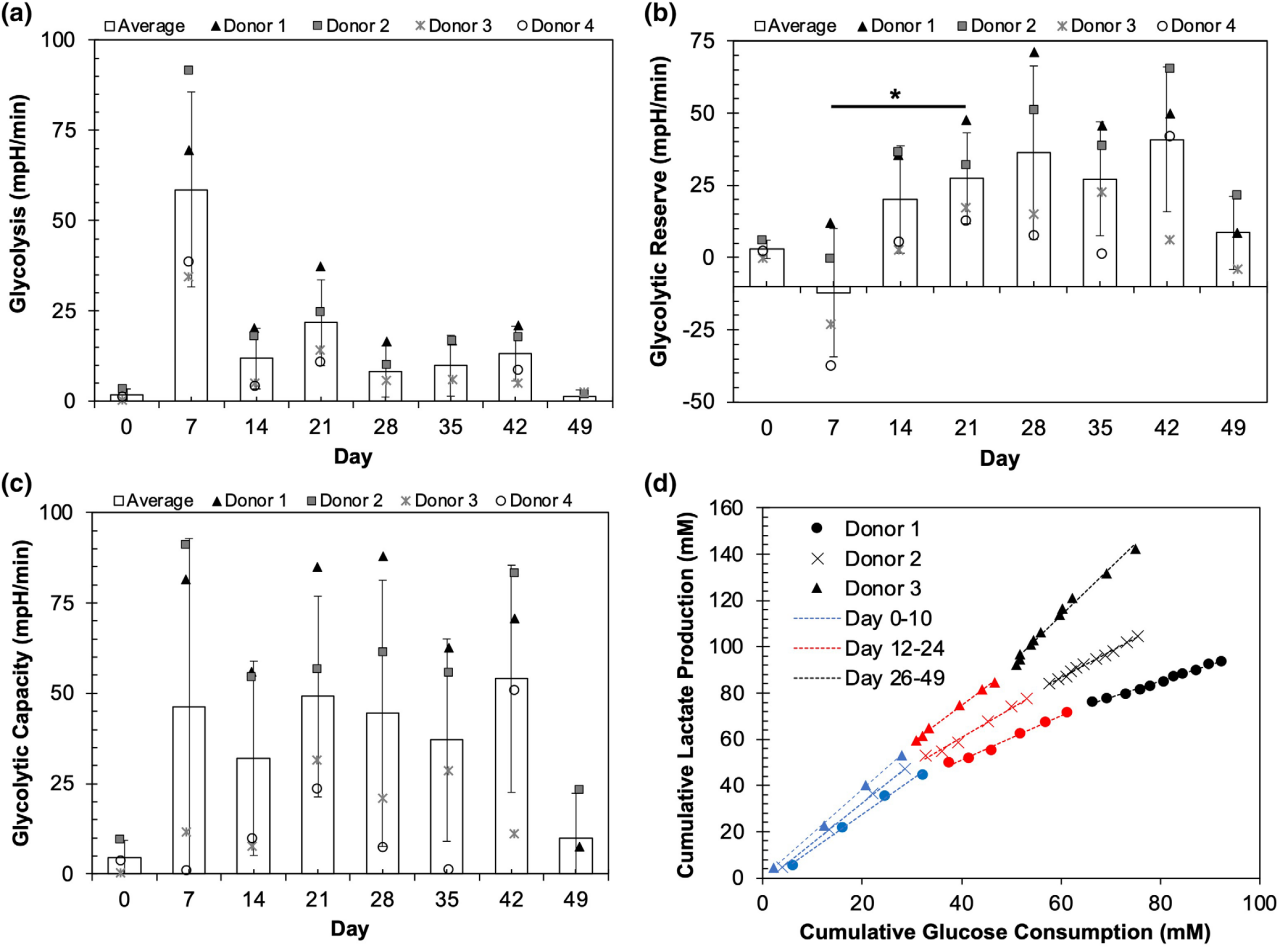
Characterization of glycolytic metabolism during NK cell expansion. (a–c) Seahorse ECAR profiles for each donor and average across all donors (white bars) for maximal glycolysis (a), glycolytic capacity (b), and glycolytic reserve (c). *p < 0.05 via a mixed effects model with Tukey's multiple comparisons tests. (d) Cumulative lactate production versus cumulative glucose consumption measured using the YSI 2950D‐3 Biochemistry Analyzer. Trendlines describe rate of lactate production relative to glucose consumption and are sectioned by time periods, where blue depicts timepoints from day 0 to day 10, after which a change in rate occurs in donors 1 and 2, as seen in red. Growth phase 2 begins after day 26 and is depicted in black. The consumption rate of glucose for Donor 4 was low and noisy, so is not plotted.

To assess NK cell glycolytic metabolism, basal glycolysis, glycolytic capacity, and glycolytic reserve were measured from the extracellular acidification rate (ECAR) (Figure 3a–c) at the basal level, maximal state after addition of glucose, and maximal state after the addition of oligomycin. The glycolytic rate showed a similar trend as oxidative mitochondrial metabolism–an increase from day 0 to day 7, a relatively steady level for the next six weeks, then a drop at the final timepoint (day 49) (Figure 3a–c).

We complemented ECAR analysis with enzymatic measurement of glucose and lactate in the culture supernatant. Cumulative glucose consumption and lactate production were calculated using material balances to account for medium changes, then plotted (Figure 3d). From these measurements, the stoichiometric conversion of glucose to lactate in different stages of expansion was determined via linear regression (Table S2). In growth phase 1, the ratio ranged from 1.5 to 1.9 mol lactate/mol glucose for all donors, as compared to the maximum conversion potential of 2.0 mol lactate/mol glucose in glycolysis. For donor 3, which maintained a faster growth rate in growth phase 2 compared to the other donors, high glucose to lactate conversion was maintained throughout culture, while the other donors showed a decrease in the conversion ratio (0.7 and 1.1 mol lactate/mol glucose for donors 1 and 2, respectively) as the culture proceeded to growth phase 2. Decreased lactate production was accompanied by an increase in the ratio of cumulative glutamine consumption to glucose consumption (Figure S2, Table S3). The slow growth rate in donor 4 and low glucose consumption in the last period rendered the measurement of glucose and lactate too noisy for calculation of conversion.

### 3.3 Functional analysis of NK cells during expansion

**FIGURE 4:**
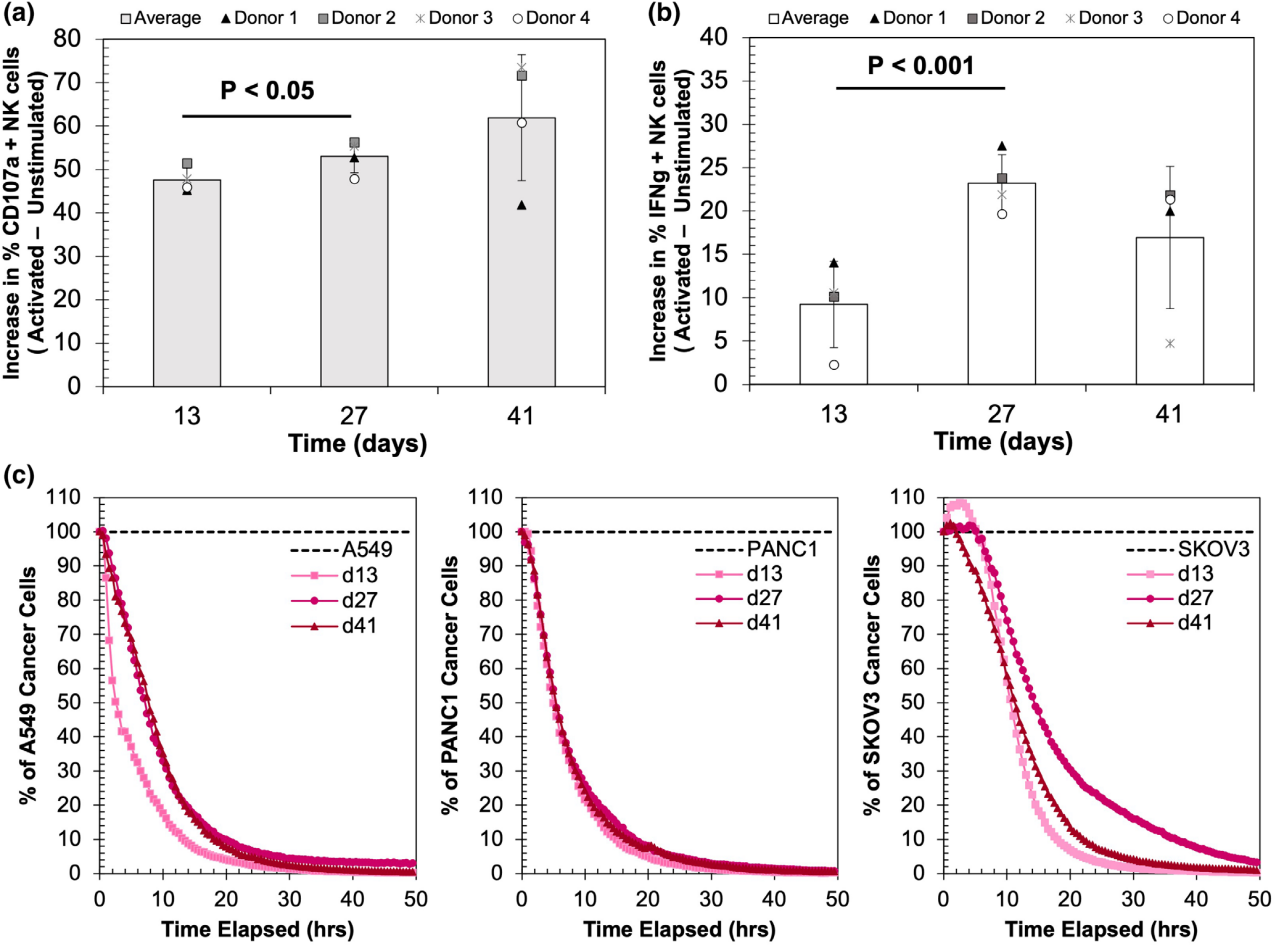
Degranulation and tumor cell killing assays to assess cytotoxicity potential of expanded NK cells. NK cells at select timepoints during the periodic restimulation were incubated at a 1:1 ratio with or without naïve K562 cells for 3.5 h. Flow cytometry was used to analyze the percent of live CD56^+^ NK cells expressing CD107a (a) and IFN‐γ (b) at days 13, 27, and 41 after incubation with K562 cells relative to NK cells incubated alone, which are referred to as “unstimulated”. Results from each donor and average across all donors are shown at each timepoint. Statistical analysis was determined via ANOVA with Tukey's multiple comparisons tests. (c) Target cell killing by donor 1 NK cells over 50 h after the start of co‐culture, as measured by the IncuCyte Live Cell Analysis System. Cytotoxicity was measured against three target cell lines (A549, PANC1, and SKOV3) expressing NucLight Red fluorescent protein at a 10:1 (NK to cancer cell) ratio at days 13, 27, and 41 of NK cell expansion. The percentage of target cancer cells at each timepoint was determined by normalizing the remaining live target cancer cells to the live cells in the target cell‐only control group.

The ability of the expanded NK cells to kill cancer cells was evaluated using the IncuCyte Live Cell Analysis System at three timepoints during the expansion process: day 13 (growth phase 1), day 27 (transition between growth phases 1 and 2), and day 41 (growth phase 2). Killing kinetics are shown in Figure 4c (donor 1) and Figure S3A–C (donors 2–4). To provide a quantitative comparison of cytotoxicity across timepoints, the percentage of tumor cells killed by hour 10 of the assay was determined for each donor at each expansion timepoint (Figure S3D). The results demonstrated slower NK cell killing kinetics at day 27 and day 41 relative to day 13 for all donors using A549 and SKOV3 as the target cells, except in the case of donor 3 with SKOV3 target cells. When PANC1 was used as the target cell, three out of four donors showed no difference in killing rate for NK cells of different stages, while for donor 4, late‐stage cells again showed slower killing. It is worth noting that PANC1 cells were killed faster by NK cells than the other two target cells. Overall, these results indicate that growth phase 2 may be associated with changes in cytotoxic function.

**FIGURE 5:**
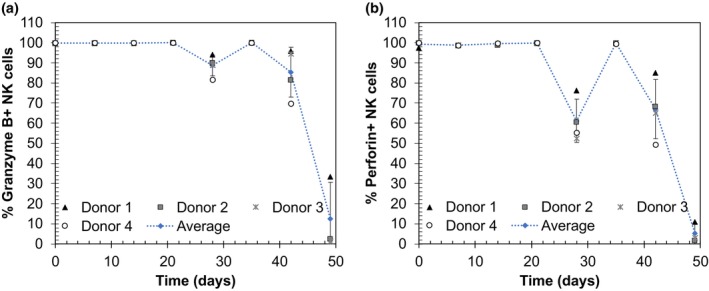
Assessment of cytotoxic functionality by flow cytometry. Percentages of CD56^+^ NK cells that express granzyme B (a) and perforin (b) prior to each re‐stimulation.

Upon stimulation by target cells, NK cells undergo degranulation and upregulate IFN‐γ expression. The degranulation assay identifies NK cells that release perforin and granzyme in response to stimuli by assessing the surface level of CD107a (LAMP1), an intracellular lysosomal membrane protein that is mobilized to the NK cell surface upon release of cytotoxic granules.^28^ Similarly, upon stimulation NK cells release IFN‐γ that sensitizes K562 cells to perforin‐dependent lysis.^29^ The capacity of NK cells to be stimulated by K562 cells was assessed by comparing the percentage of CD107a or IFN‐γ positive cells with and without target cell activation. The percentages of CD107a + and IFN‐γ + NK cells increased from day 13 to day 27 and maintained similar levels at day 41, though donor 3, which maintained higher levels of growth, showed a large drop in IFN‐γ + NK cells at day 41 (Figure 4). Weekly analysis of intracellular perforin and granzyme B expression immediately before re‐stimulation showed nearly all NK cells in growth phase 1 expressed perforin and granzyme B (Figure 5a,b). In growth phase 2 (day 28–49), the percent expression began fluctuating, and a large reduction in granzyme B and perforin expressing cells was seen at day 49, coinciding with the decrease in metabolic activities. Collectively, the shift in growth kinetics coincides with changes in perforin and granzyme expression, but neither degranulation nor cytokine production are reduced upon the switch to slower growth.

### 3.4 Transcriptomic analysis of NK cells in early and late‐stage culture

**FIGURE 6:**
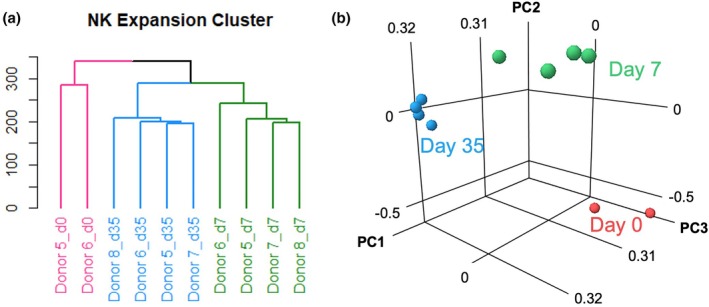
RNA sequencing analysis of NK cells in different growth phases. (a, b) Hierarchical clustering (a) and principal component analysis (b) of RNA sequencing data for donors 5–8, which include non‐activated samples (day 0) from donors 5–6 and activated samples from growth phase 1 (day 7) and growth phase 2 (day 35) from donors 5–8.

RNA‐seq was performed on NK cells from four additional donors (donors 5–8) at day 0 (pre‐stimulation), day 7 (growth phase 1), and day 35 (growth phase 2). Consistent with the first four donors, these donors exhibited a shift to slower cell growth during the culture (Figure S4). Hierarchical clustering (Figure 6a) and principal component analysis (Figure 6b) both show that samples clustered together by timepoint rather than by donor. A significant number of genes were differentially regulated in the same direction at days 7 and 35 relative to day 0, with a larger number of genes down‐regulated (766) than up‐regulated (442) (Figure S5). There were a small number of genes that showed differential expression at only one of the timepoints (93 and 85 at day 7 and day 35, respectively). An even smaller number of genes were differentially expressed in opposite directions at days 7 and 35 (Figure S5).

**FIGURE 7:**
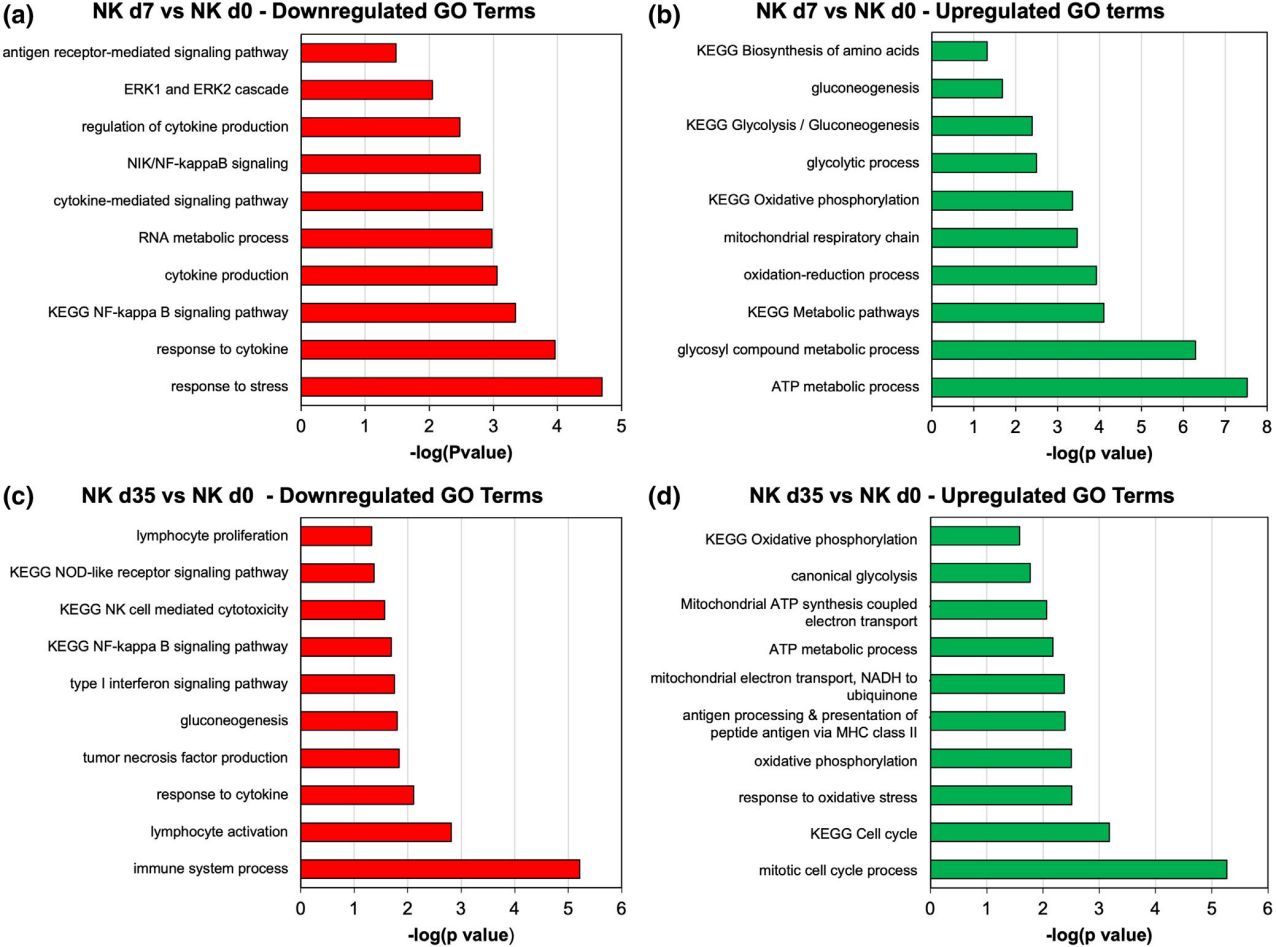
Enriched GO terms of early and late culture stage NK cells. (a, b) Gene ontology analysis showing key downregulated (a) terms and upregulated (b) terms in Day 7 RNA sequencing samples (compared to day 0). (c‐d) GO analysis showing key downregulated (c) and upregulated (d) terms in Day 35 RNA sequencing samples (compared to day 0).

Gene ontology (GO) analysis of transcripts which were downregulated at day 7 compared to day 0 revealed an enrichment of GO terms related to cytokine‐mediated pathways, cytokine production and response to stress/cytokine (Figure 7a). Pathways which were downregulated at day 35 compared to day 0 included lymphocyte proliferation, cell‐mediated cytotoxicity, gluconeogenesis, and response to cytokine stimulation (Figure 7c). GO terms related to energy metabolism such as gluconeogenesis, the glycolytic process, and oxidative phosphorylation were upregulated at day 7 compared to day 0 (Figure 7b). The energy metabolism pathways continued to be upregulated in the day 35 to day 0 comparison, including mitochondria‐related metabolism such as oxidative phosphorylation and the electron transport chain (Figure 7d). Expression dynamics of transcripts associated with NK cell activation and function as well as GO‐enriched transcripts in key functional categories such as metabolism, cell cycle, and NFκB signaling pathways are shown in Figure S6.

To directly assess changes in genes associated with key metabolic pathways in early and late‐stage culture, the RNA sequencing results were mapped onto pathway diagrams for glycolysis, the TCA cycle, the malate–aspartate shuttle, and part of the pentose phosphate pathway (Figure S7). Genes associated with glycolysis, including key glycolytic enzymes, were generally upregulated after feeder cell stimulation at day 7 and decreased at day 35 to levels that were still higher than day 0. Similarly, enzymes associated with the TCA cycle were upregulated from day 0 to day 7, but the transcript levels were largely maintained at similar levels between day 7 and day 35 (Figure S7).

## 4 DISCUSSION

In vitro expanded NK cells have the potential to become off‐the‐shelf therapies for the treatment of cancers and other diseases. To achieve that goal, it is critical to develop a biomanufacturing process that expands donor cells to a sufficiently large number. The development of engineered K562 feeder cells expressing membrane‐bound IL‐21 and 41BBL (K562‐mbIL21‐41BBL) has enabled large‐scale expansion of NK cells derived from peripheral blood, with K562‐mbIL21‐41BBL cells achieving 1 × 10^8^‐fold NK cell expansion by day 42 in culture.^17^ Hence, K562‐mbIL21‐41BBL feeder cell‐induced NK cell expansion can be exploited for scale up of cell biomanufacturing. This study characterized the effects of repeated stimulation with K562 cells expressing 41BBL and mbIL21 on kinetics of NK cell growth, function, and metabolism, as these metrics ultimately impact the efficacy of the cell‐based therapy. While there has been some characterization of growth kinetics, receptor expression, and functionality of NK cells during long‐term expansion,^17^, ^18^, ^30^, ^31^, ^32^ the integrated analysis of metabolic behavior with proliferative and functional capacity has not been demonstrated beyond 21 days. Detailed kinetic analysis can reveal subtle shifts in NK cell metabolism and phenotype. Furthermore, these studies are critical to determine if and at what point achieving significant expansion compromises NK cell function, thus impacting efficacy of the final therapeutic product.

Using serial stimulation with feeder cells, we demonstrated NK cell expansion of ~2 × 10^8^‐fold over 42 days, a level similar to that reported previously.^17^ Quantitative analysis of the growth kinetics showed that a faster growth period, which yielded 1 × 10^5^‐fold expansion in 21 days, was followed by a shift to a period of slower growth, with a decrease in doubling time and accompanied by a slight decrease in viability at later timepoints. Our study further revealed that the change in growth rate around day 21–26 in this study was accompanied by an accumulation of cells in the G0/G1 cell cycle phase. Consistent with the change in growth kinetics, GO analysis also showed downregulation of biological processes related to lymphocyte proliferation at day 35. Collectively, our results support that donor‐derived NK cells can expand for 8 weeks but diminish in proliferative capacity after 3–4 weeks. In this study, expansion was not continued past day 49. However, Streltsova et al. expanded NK cells 4 × 10^6^‐fold from single cell clones over 98 days by stimulating with K562 cells engineered with membrane‐bound IL‐21 at a lower frequency, only at day 0 and day 42, indicating NK cells can remain in ex vivo culture for even longer durations of time.^20^

NK cell effector functions entail both direct cell–cell cytotoxicity and cytokine production. Expanding NK cells demonstrated cytotoxicity against cancer cell targets in both growth phase 1 and 2, though faster killing was observed in growth phase 1 (day 13) for two of the three cancer lines (A549 and SKOV3). GO analysis echoed these results, with the NK‐cell mediated cytotoxicity functional class showing downregulation at day 35. Wang et al. showed a similar trend in NK cell killing activity using a calcein release assay, with cytotoxicity peaking at weeks 3–5 before decreasing after 6 weeks.^18^ Killing rates stayed consistent over time against PANC1 cells for donors 1–3. This could be due to the use of a high effector to target cell ratio (10:1), which could mask subtle differences in killing efficacy,^33^ particularly for cell lines that exhibit faster rates of killing by NK cells. Thus, lower effector to target ratios may be needed to fully characterize shifts in NK cell function against a tumor target of interest. Flow cytometry results indicated that degranulation, as evidenced by CD107a expression, remains consistent throughout expansion, though variability in intracellular levels of perforin and granzyme in growth phase 2 suggest a possible mechanism for observed shifts in cytotoxicity. Cytokine production during the degranulation assay was assessed via expression of IFN‐γ, which is produced by primed NK cells and sensitizes K562 cells to perforin‐dependent lysis.^29^ The results showed that IFN‐γ production did not decrease during extended culture. Collectively, our results demonstrated attenuated cytotoxicity but not diminished cytokine production in late stages of culture.

Metabolism, effector functions, and cell growth are closely linked. K562‐mbIL21‐expanded NK cells at day 21 have been demonstrated to exhibit Warburg metabolism, operating at maximum glycolytic capacity, primarily due to STAT3 signaling via IL‐21.^31^ Furthermore, recent studies have shown that NK cell metabolism is intrinsically linked to effector function and IFN‐γ production.^34^, ^35^, ^36^, ^37^ Seahorse assays showed a large increase in glycolysis and oxidative phosphorylation, which was confirmed by expression levels of key metabolic enzymes in the RNA sequencing results, upon the transition to proliferation following initial feeder cell stimulation, reflecting the surge in energy demand and subsequent supply needed to sustain cell proliferation. GSEA results from cord blood‐derived NK cells expanded with K562 engineered with CD48, 41BBL, and membrane‐bound IL‐21 at day 15 show similar enrichment of glycolysis and oxidative phosphorylation.^38^ Rapidly proliferating cells exhibit elevated aerobic glycolysis, consuming more glucose and converting a large portion of glucose to lactate.^39^ Aerobic glycolysis enables rapid ATP production, as well as more efficient biosynthesis and accumulation of biomass to support rapid proliferation.^39^ Quantification of glucose consumption and lactate production via enzymatic assays demonstrated that in the first stage of growth, the conversion of glucose to lactate was near the maximum possible level of 2 mol lactate / mol glucose, suggesting that the vast majority of glucose consumed was converted to lactate, with only a small portion entering the citric acid (TCA) cycle and oxidative metabolism.

Glycolytic and mitochondrial metabolism levels were sustained in Seahorse assays throughout extended culture, with the exception of the final timepoint (day 49). RNA sequencing results showed that glycolytic enzymes remained upregulated in growth phase 2, though at lower levels than growth phase 1, whereas enzymes associated with the TCA cycle maintained similar levels of upregulation in both growth phases. However, a decrease in the portion of glucose converted to lactate was observed in the second growth phase, indicating a reduction in aerobic glycolysis, with more glucose being metabolized through oxidative metabolism. This occurred alongside increased consumption of glutamine, which is typically the second highest consumed carbon source for cells in culture. Glutamine plays a critical anaplerotic role in replenishing metabolic intermediates of the TCA cycle, such as citrate and oxaloacetate, that have been utilized for biosynthesis.^40^, ^41^ Collectively, increased glutamine consumption with reduced conversion of glucose to lactate supports a reduction in aerobic glycolysis. As expected, the metabolic transition to reduced aerobic glycolysis coincided with the slowdown in growth and may also correlate to the observed attenuation in cytotoxicity in growth phase 2, as a study by Chang et al. found that glycolysis is key for T cell effector function.^42^ The fact that a reduced rate of glucose to lactate conversion was not observed in donor 3, which also did not exhibit significantly slowed growth in extended culture, further underscores the metabolic contribution of aerobic glycolysis to proliferation in expanding NK cells. Taken together, these results are supportive of a metabolic basis for reduced growth and dysfunction in NK cells in late stages of culture. Metabolic modulation through culture conditions or medium design represents a promising avenue for optimization. The effects of altering glucose levels on NK cell growth and functionality remain largely unexplored, warranting further investigation to advance this field.

Cytotoxic T cells and NK cells can progressively lose proliferative potential, cytokine production, and cytotoxic function due to exhaustion and/or senescence. There has been significant effort toward investigation of NK cell exhaustion as it relates to the treatment of tumors; however, little is known about possible exhaustion of NK cells cultivated in vitro. Although poorly characterized, decreases in IFN‐γ, CD107a, perforin, and granzyme, and cytotoxicity, some of which were observed in growth phase 2, are typically attributed to exhaustion.^43^ *CDKN2A* (cyclin‐dependent kinase inhibitor 2A), which plays a role in senescence and aging,^44^ is upregulated in the day 35 samples compared to day 7 and day 0 samples (data not shown). In addition, decreased proliferation has been associated with NK cell senescence.^43^, ^45^ These results, coupled with the rapid drop of metabolic activities, perforin and granzyme B expression, and cytotoxicity at the end of cell expansion (Day 49), suggest the cells may become exhausted or senescent upon repeated in vitro stimulation, which has implications for therapeutic efficacy.

## 5 CONCLUSION

Collectively, our results demonstrated that achieving on the order of 10^8^‐fold expansion of NK cells from a single donor via repeated feeder cell stimulation is accompanied by shifts in growth kinetics, metabolism, and function that must be carefully characterized to determine the appropriate degree of expansion for desired product quality. A better understanding of the cause of this growth and metabolic transition will help determine whether such a transition is of any concern for therapeutic applications, and whether these issues can be mitigated by engineering the bioprocess. It may be true that in large‐scale biomanufacturing of NK cells, maintaining metabolically active and cytotoxic cells is preferred over reaching the full expansion potential of these cells. It is noted that even before transitioning to slower growth, NK cells have achieved roughly 10^6^‐fold expansion, which would be sufficient to produce hundreds of doses. We also contend that the NK cell expansion process described in this study is not fully optimized and can be enhanced with some process intensification. As more NK cells enter clinical applications, transforming NK cell expansion into a robust process will be critical to ensure the success of cell therapies.

## AUTHOR CONTRIBUTIONS

**Jennifer One:** Conceptualization, Investigation, Formal analysis, Methodology, Validation, Visualization, Writing – original draft, Writing – review & editing. **Janani Narayan:** Data curation, Formal analysis, Investigation, Validation, Visualization, Writing – review & editing. **Frank Cichocki:** Conceptualization, Supervision, Writing – review & editing. **Wei‐Shou Hu:** Conceptualization, Supervision, Writing – review & editing. **Samira M. Azarin:** Project administration, Supervision, Conceptualization, Funding acquisition, Writing – review & editing.

## CONFLICT OF INTEREST STATEMENT

The authors declare no conflicts of interest.

## Supporting information


**Data S1.** Supporting Information.

## Data Availability

The datasets used and/or analyzed in the current study are available from the corresponding author upon reasonable request. RNA‐seq data generated in this manuscript will be publicly available from the Gene Expression Omnibus (GSE311384) from November 24, 2026.
