# Supplementary material for: Dynamics of natural killer cell function upon recurrent stimulation
Source: Biotechnol Prog. 2026 Jan 13;42(2):e70100. doi: 10.1002/btpr.70100 (PMC13055136; doi:10.1002/btpr.70100)
Supplement: Supplementary file 1 — Data S1. Supporting Information. [file BTPR-42-e70100-s001.pdf]

Dynamics of Natural Killer Cell Function upon Recurrent Stimulation

Jennifer One, Janani Narayan, Frank Cichocki, Wei-Shou Hu, and Samira M. Azarin

Supplementary Materials:

Supplementary Tables S1-S3

Supplementary Figures S1-S7

Table S1. Detailed information for antibodies used for flow cytometry.

Target antigen	Antibody species	Vendor	Clone or product number	ul/Test	Panel
CD3	Human	BioLegend	317330; BV785 Clone: OKT3	3ul	IC, DG
CD56	Human	BioLegend	318318; PE/Cy7 Clone: HDCD56	3ul	IC, DG
CD107a	Human	BioLegend	PE/Cy5.5 Clone: H4A3	3ul	DG
Granzyme B	Human/mouse	BioLegend	372206; FITC Clone: AQ16A02	5ul	IC
IFNγ	Human	BioLegend	BV650 Clone: B27	3ul	DG
Perforin	Human	BioLegend	353304; PE Clone: B-D48	5ul	IC

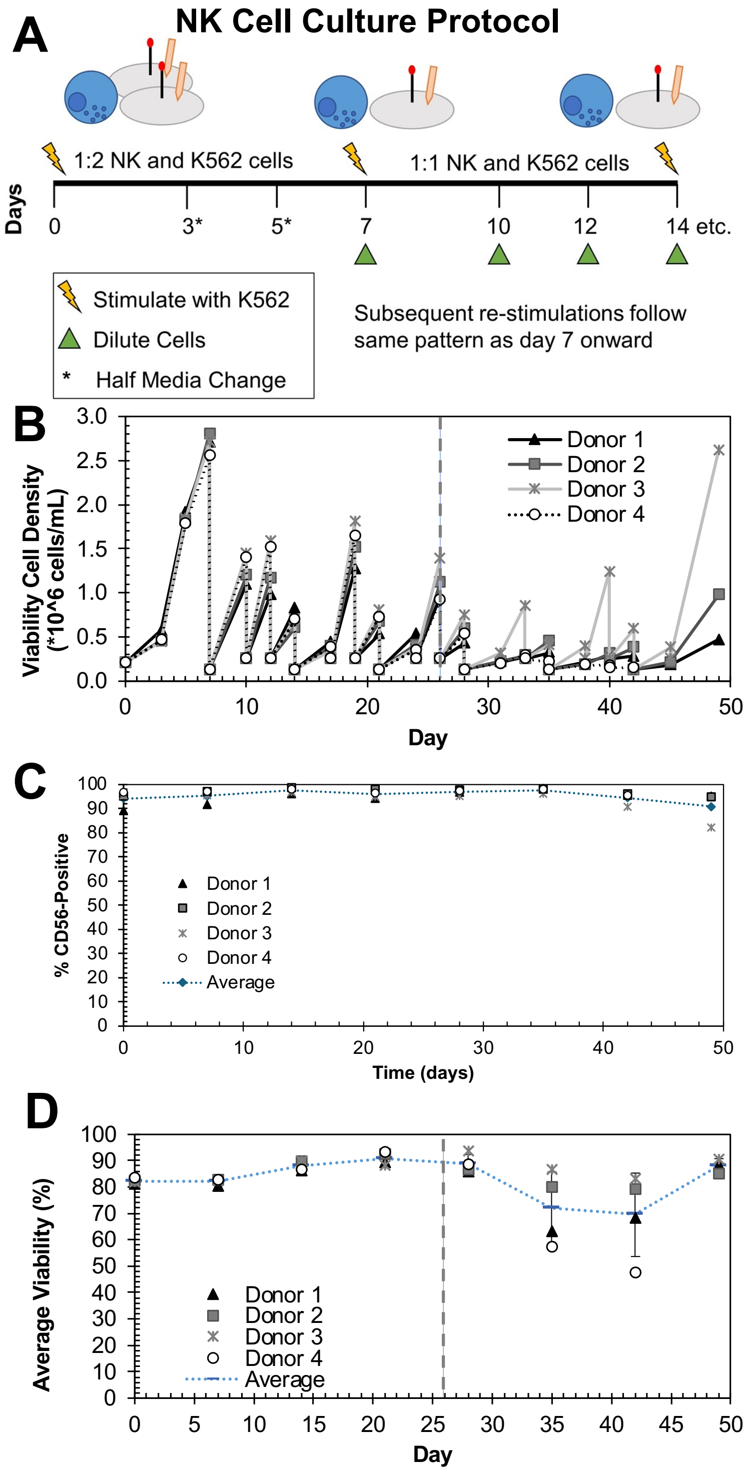

Figure S1. NK cell expansion process. (A) NK cell culture protocol. (B) Viable cell density of the NK cell culture over time for each donor. (C) Percentage of CD56-positive cells (or each donor as well as the average across donors) evaluated every 7 days using flow cytometry. (D) Average percent viability (for each donor as well as the average across donors) evaluated every 7 days, immediately prior to addition of K562 feeder cells.

Table S2. Molar ratio of lactate production to glucose consumption as determined by linear regression. Slopes and corresponding coefficient of determination are presented for each sectioned culture time for Donors 1-3.

	Culture Time	Slope	R^2
**Donor 1**	Day 0-10	1.51	0.996
	Day 12-24	0.95	0.989
	Day 26-49	0.71	0.992
**Donor 2**	Day 0-10	1.73	1.000
	Day 12-24	1.27	0.992
	Day 26-49	1.14	0.994
**Donor 3**	Day 0-10	1.90	0.999
	Day 12-24	1.60	0.996
	Day 26-49	2.08	0.986

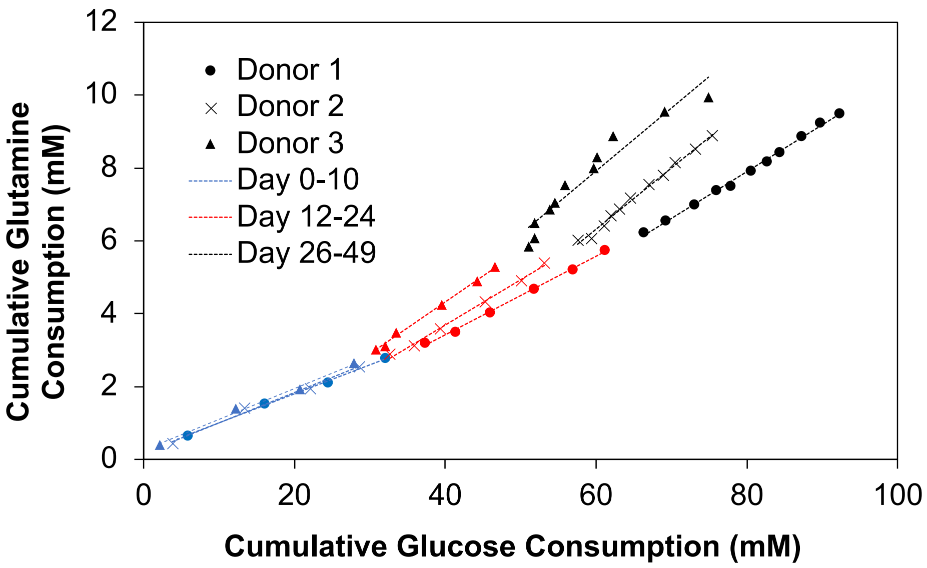

Figure S2. Ratio of cumulative glutamine consumption to glucose consumption during NK cell expansion. Cumulative glutamine consumption versus cumulative glucose consumption measured through enzymatic assay using the YSI analyzer. Donors are distinguished by shape, where donor 1 is denoted by circles, donor 2 by crosses, and donor 3 by triangles. Trendlines describe rate of glutamine consumption relative to glucose consumption and are sectioned by time periods, where blue depicts timepoints from day 0 to day 10, after which a change in rate occurs in donors 1 and 2, as seen in red. Growth phase 2 begins after day 26 and is depicted in black. The consumption rate of glucose for Donor 4 was low and noisy, hence is not plotted.

Table S3. Molar ratio of glutamine consumption to glucose consumption as determined by linear regression. Slopes and corresponding coefficient of determination are presented for each sectioned culture time for Donors 1-3.

	Culture Time	Slope	R^2
**Donor 1**	Day 0-10	0.08	0.997
	Day 12-24	0.11	0.998
	Day 26-49	0.13	0.996
**Donor 2**	Day 0-10	0.08	0.988
	Day 12-24	0.12	0.995
	Day 26-49	0.17	0.994
**Donor 3**	Day 0-10	0.08	0.994
	Day 12-24	0.14	0.997
	Day 26-49	0.17	0.931

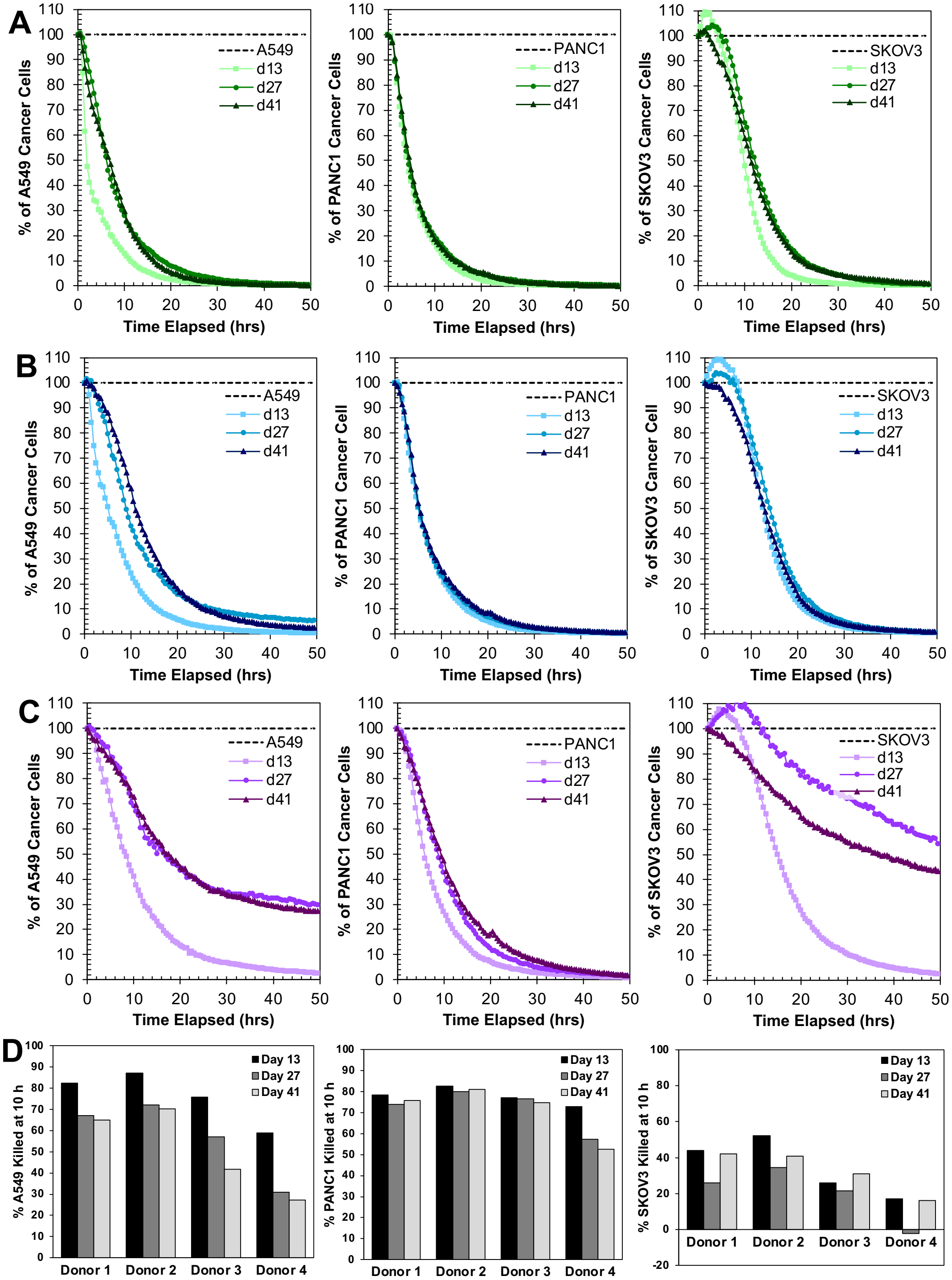

Figure S3. Characterization of expanded NK cell killing kinetics. (A-C) NK cells from donors 2 (A), 3 (B) and 4 (C) were incubated with A549 cells (left), PANC-1 cells (center), or SKOV-3 cells (right) expressing NucLight Red fluorescent protein at a 10:1 (NK:cancer cell) ratio. The percentage of target cancer cells at each timepoint was determined by normalizing the remaining live target cancer cells to the live cells in the target cell-only control group using the IncuCyte Live Cell Analysis System. Donor 1 data is shown in Figure 4C. (D) Percentage of target cells killed at hour 10 of the IncuCyte assay for each donor, expansion timepoint and target cell line.

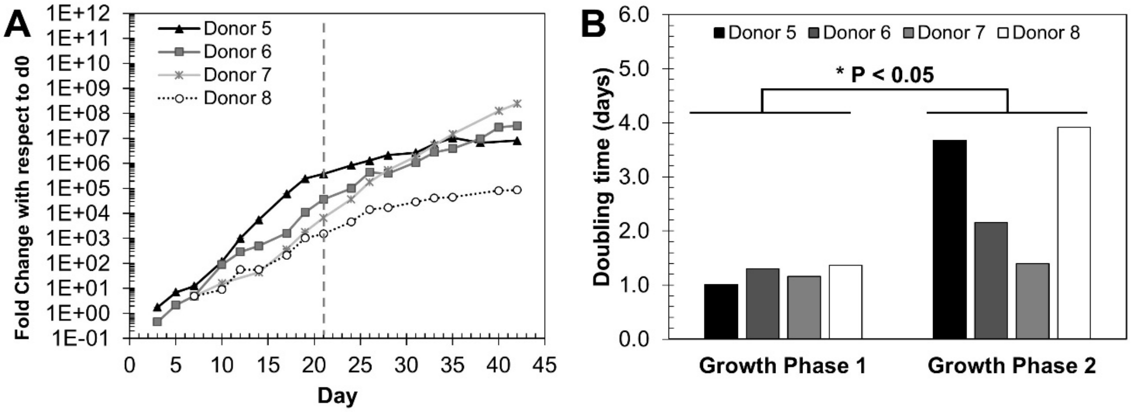

Figure S4. Growth kinetics of NK cells used in transcriptome analysis. (A) Cumulative fold change over time for Donors 5-8; dashed line indicates the transition to the slower growth phase at day 21. (B) Doubling time in days for growth phase 1 (prior to day 21) and growth phase 2 (after day 21 for each donor). *P < 0.05 (paired t test).

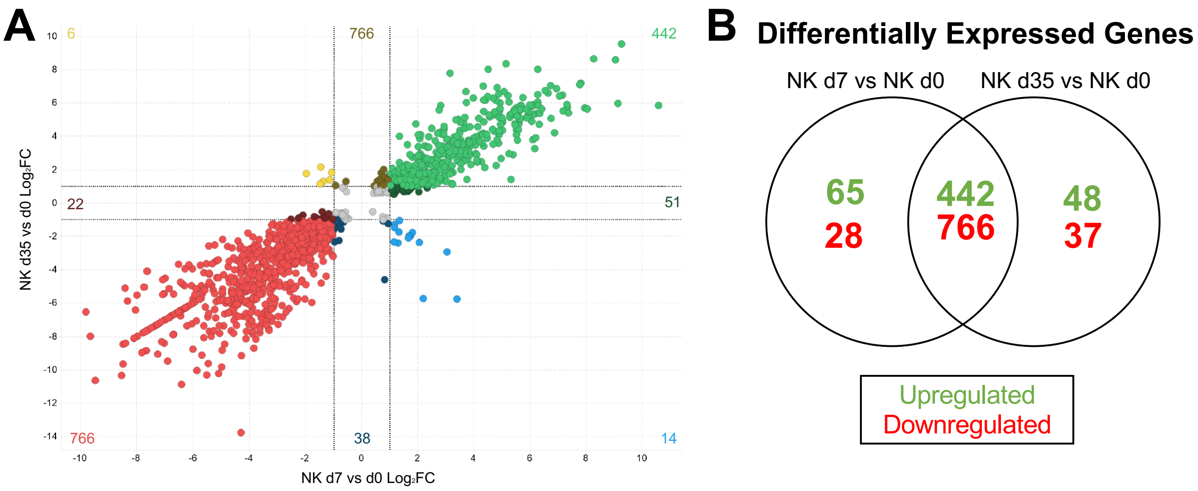

Figure S5. Differential gene expression analysis of expanded NK cells. (A) Quadrant analysis showing the pairwise comparison of activated NK cells at day 7 (growth phase 1) and day 35 (growth phase 2), both normalized to day 0. Plot shows genes that are commonly upregulated (green) and downregulated (red) between the pairwise comparisons (d7 vs. d0 and d35 vs. d0). Genes that were differentially expressed in opposite directions at days 7 and 35 are shown in blue and yellow. Pairwise comparison criteria for differentially expressed genes included: log2 fold change expression >1 or <-1, p-value < 0.05, and FDR < 0.05. (B) Venn diagram of the differentially expressed genes for the pairwise comparisons showing genes that were differentially regulated in the same direction at both timepoints and genes that were only differentially expressed at one of the time points.

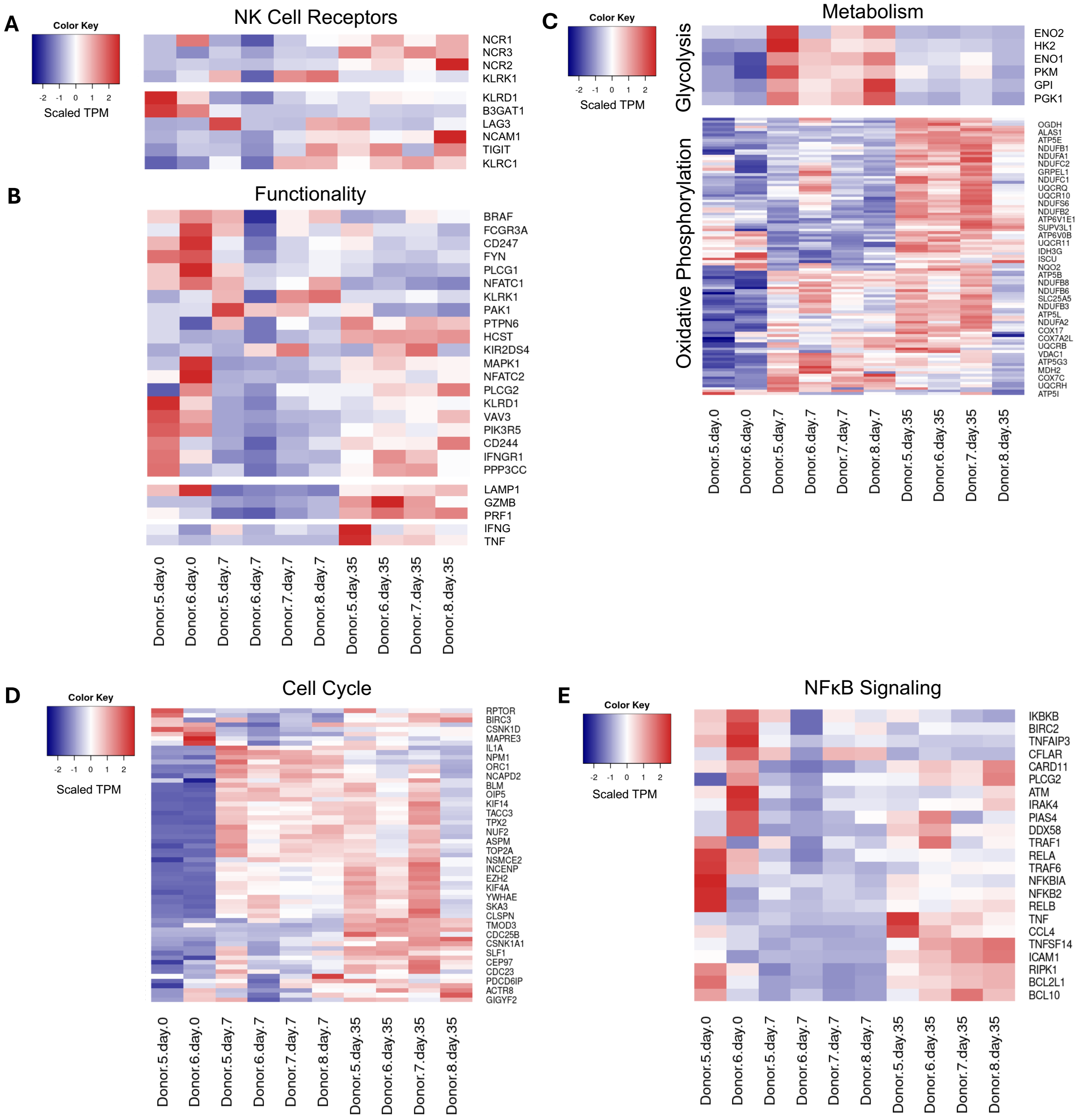

Figure S6. Transcript expression of key NK cell markers and enriched genes. Gene names are presented to the right of the heatmap and samples are on the bottom. The color key shows scaled TPM values, with blue corresponding to low expression and red corresponding to high expression. (A) Heatmap of transcript expression for activating and inhibitory receptors as well as CD56 (*NCAM1*) and CD57 (*B3GAT1*). (B) Expression of transcripts related to functionality, including differentially expressed genes from the KEGG NK cell mediated cytotoxicity functional class as well as transcripts relevant for degranulation and cytokine production. (C) Expression of transcripts related to metabolism functional classes, including glycolysis (GO:0061621) and oxidative phosphorylation (GO:0006119). (D,E) Transcripts related to the (D) GO cell cycle (GO:0007049) and (E) NFKB signaling (GO:0007249) functional classes, which were elevated in over-representation analysis.

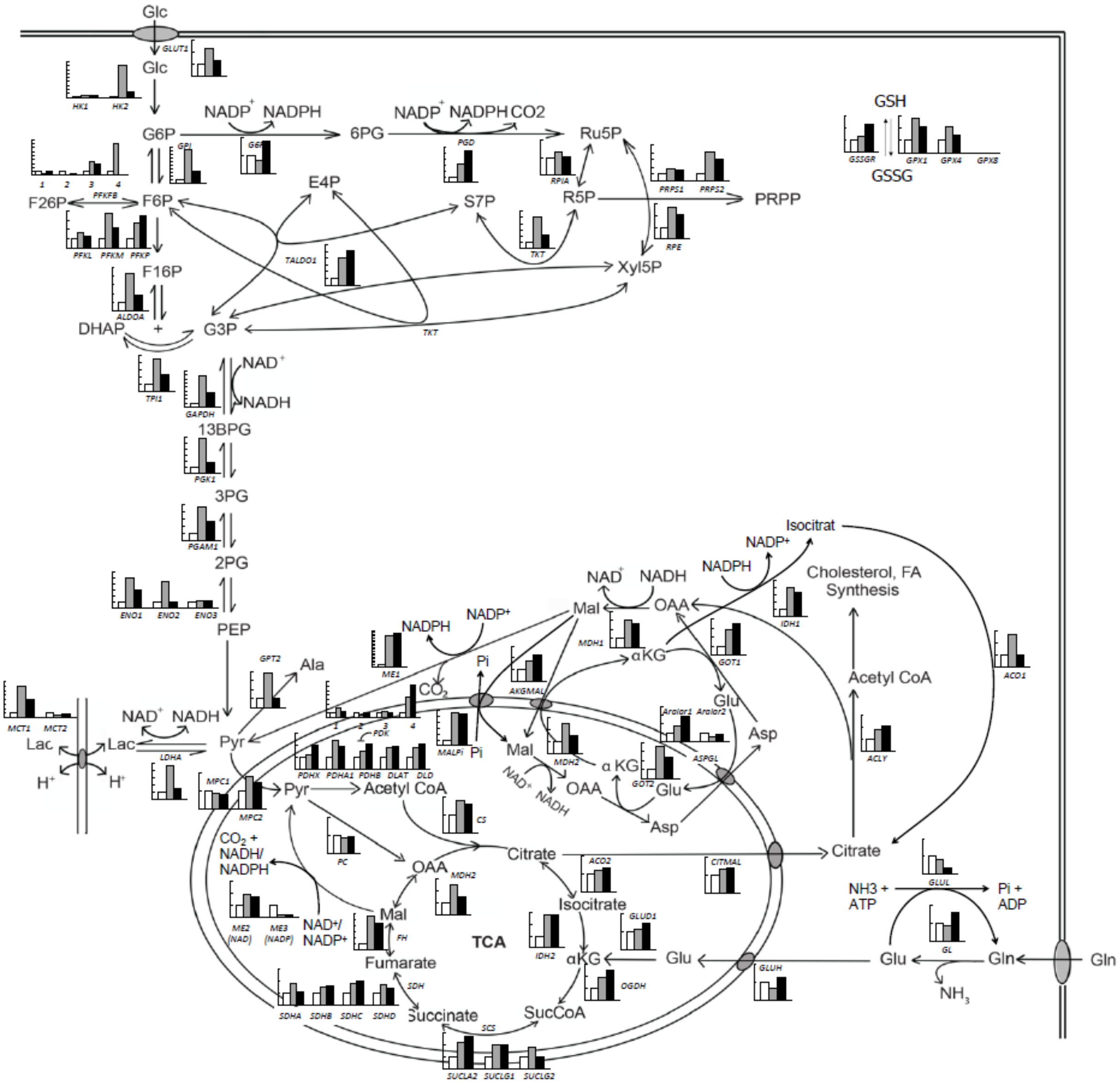

Figure S7. Mapping of RNA sequencing results onto key metabolic pathways. Bar graphs indicate relative TPM value expression compared to the control (Day 0). Each TPM value was the average of all the donors for that timepoint. Results were obtained from Donors 5-6 for Day 0 (white bars) and Donors 5-8 for Day 7 (grey bars) and Day 35 (black bars).
